# Neurologic adverse events associated with smallpox vaccination in the United States – response and comment on reporting of headaches as adverse events after smallpox vaccination among military and civilian personnel

**DOI:** 10.1186/1741-7015-4-27

**Published:** 2006-11-10

**Authors:** Walter R Schumm

**Affiliations:** 1 School of Family Studies and Human Services, College of Human Ecology, Justin Hall, Kansas State University, 1700 Anderson Avenue, Manhattan, KS, USA 66506-1403

## Abstract

**Background:**

Accurate reporting of adverse events occurring after vaccination is an important component of determining risk-benefit ratios for vaccinations. Controversy has developed over alleged underreporting of adverse events within U.S. military samples. This report examines the accuracy of adverse event rates recently published for headaches, and examines the issue of underreporting of headaches as a function of civilian or military sources and as a function of passive versus active surveillance.

**Methods:**

A report by Sejvar et al was examined closely for accuracy with respect to the reporting of neurologic adverse events associated with smallpox vaccination in the United States. Rates for headaches were reported by several scholarly sources, in addition to Sejvar et al, permitting a comparison of reporting rates as a function of source and type of surveillance.

**Results:**

Several major errors or omissions were identified in Sejvar et al. The count of civilian subjects vaccinated and the totals of both civilians and military personnel vaccinated were reported incorrectly by Sejvar et al. Counts of headaches reported in VAERS were lower (n = 95) for Sejvar et al than for Casey et al (n = 111) even though the former allegedly used 665,000 subjects while the latter used fewer than 40,000 subjects, with both using approximately the same civilian sources. Consequently, rates of nearly 20 neurologic adverse events reported by Sejvar et al were also incorrectly calculated. Underreporting of headaches after smallpox vaccination appears to increase for military samples and for passive adverse event reporting systems.

**Conclusion:**

Until revised or corrected, the rates of neurologic adverse events after smallpox vaccinated reported by Sejvar et al must be deemed invalid. The concept of determining overall rates of adverse events by combining small civilian samples with large military samples appears to be invalid. Reports of headaches as adverse events after smallpox vaccination appear to be have occurred much less frequently using passive surveillance systems and by members of the U.S. military compared to civilians, especially those employed in healthcare occupations. Such concerns impact risk-benefit ratios associated with vaccines and weigh against making vaccinations mandatory, without informed consent, even among military members. Because of the issues raised here, adverse event rates derived solely or primarily from U.S. Department of Defense reporting systems, especially passive surveillance systems, should not be used, given better alternatives, for making public health policy decisions.

## Background

Recently, Sejvar et al [1] reviewed the U.S. Vaccine Adverse Event Reporting System (VAERS) reports from 64,600 civilians, mostly health care workers, and from 590,400 Department of Defense (DoD) employees who had been vaccinated against smallpox. Their stated objective was "To determine rates and describe the clinical features of neurologic events associated with smallpox vaccination." The civilians had been vaccinated by the Department of Health and Human Services (DHHS) while the DoD employees had been vaccinated within the U.S. military medical system. The VAERS reports had been submitted between December 2002 and March 2004. VAERS reports involving neurologic symptoms for 214 individuals were analyzed, 111 for DHHS vaccinees and 103 for DoD vaccinees. The most common neurologic symptom reported was headache, in 95 (44%) of the 214 cases. They concluded that "During the 2002–2004 smallpox vaccination campaign, neurologic events were generally mild and self-limited, and no neurologic syndrome was identified at a rate above baseline estimates."

Reviewing Sejvar et al [1], it is clear that they stated, as fact, that they had data from (a) 64,600 DHHS vaccinees, (b) 590,400 DoD vaccinees, and (c) a total of approximately 665,000 personnel. They also stated, as fact, that a review of all VAERS reports submitted between December 2002 and March 2004 for those 665,000 personnel yielded no more than 95 reports involving headaches. Furthermore, they presented data (see Table 2 in [1]) indicating a rate of 14.3 headaches "with or without other symptoms" per 100,000 vaccinations; that rate is clearly derived from dividing 95 headaches by 665,000 vaccinations.

The risk-benefit ratios of vaccination are very important [2-4]. Public trust depends upon accurate reporting and interpretation of research data on adverse outcomes associated with vaccination, especially for programs associated with bioterrorism, such as the smallpox vaccination campaign conducted in the United States since 2002. It is also important that public trust be maintained with respect to adverse outcomes associated with vaccination among military service members, who may be required to accept mandatory vaccinations even without informed consent.

Issues also have been raised whether rates of adverse reports among military members or DoD employees have tended to be underreported [5-7]. A variety of ideas have been proposed to account for such underreporting, which even military health experts have admitted is possible [8]. The differing age and gender compositions of civilian and military populations have been cited, as well as general health and access to good medical care [6]. Many military personnel may not be available for reporting adverse events because of pre-deployment activities [9] or because of distant geographic location with minimal access to reporting channels.

Reports of adverse reactions associated with the smallpox vaccine in a number of scientific sources [8-12] were compared to the facts alleged by Sejvar et al [1]. In particular, the rates of reported headaches were considered as a function of source and type of adverse event reporting. Several important inconsistencies were observed. However, due to the selection of different adverse events for study, it was not possible to compare most of the studies except with respect to the symptom of headache. For example, using their mixture of 665,000 civilians and military personnel, Sejvar et al [1] considered headache, limb paresthesias, dizziness/vertigo, limb pain, meningitis, Bell's palsy, limb weakness, seizures, syncope/presyncope, encephalitis, tinnitus, Guillain-Barre syndrome, dysphagia, demyelinating disease, brachial neuritis, and stroke as adverse events, whereas Grabenstein & Winkenwerder [8] considered an almost completely different set of conditions: mild, generalized vaccinia, erythema multiforme, encephalitis, acute myopericarditis, eczema vaccinatum, progressive vaccinia, and death, as adverse events among 450,923 military personnel. Relevant smallpox vaccination studies that concerned myopericarditis [13] or folliculitis [14] were not considered here.

## Type of adverse event (active versus passive) reporting

VAERS is a passive surveillance system used, in part, to help detect rates of rare but serious adverse reactions to vaccinations. However, some reports have involved active surveillance of adverse reactions to smallpox vaccine [8-10,12]. Using VAERS, Sejvar et al [1] identified 95 headaches as reported adverse events among 665,000 vaccinated individuals. Also using VAERS, Casey et al [11] identified at least 110 headache cases among 38,885 civilian recipients of smallpox vaccination. Using active surveillance of only 526 vaccinated personnel assigned to seven selected military organizations, Grabenstein & Winkenwerder [8] found 95 headaches as reported adverse events. Also using active surveillance of 680 civilian volunteers for a random single-blind trial, Frey et al [10] received reports of 294 headaches in the first six days after smallpox vaccination, with an unspecified number of new cases developing after six days (they reported the number of headaches after day 6 without indicating how many were *new *cases). With 936 mostly civilian subjects, Baggs et al [12] received 266 reports of headaches within 20 days of smallpox vaccination. In a study of 1,212 U.S. civilian DoD and military personnel who participated as first-time vaccinees (N = 439) or re-vaccinees (N = 773) in a trial of the use of electronic reporting of adverse events, Olmstead et al [9] received 317 reported incidences of headaches. Consistently, as noted in Table 1, passive event reporting systems yielded lower rates of headache as an adverse event than did active event reporting systems.

## Source of adverse event (military versus civilian) reporting

Table 1 presents the percentage of headaches reported as adverse events following smallpox vaccinations as reported across both a variety of types of reporting (passive versus active), as discussed previously, and across a variety of samples (military versus civilian compositions). Within VAERS (passive) reports, Casey et al [11] found at least 110 headaches (0.28%) among 38,885 civilians in contrast to Sejvar et al [1] who found only 95 headaches (0.01%) reported among their mixture of 665,000 civilian and military personnel. Even if nearly all of the headaches reported by Sejvar et al [1] were military personnel related, the reporting rate in VAERS for headaches (< 0.02%) would have been much lower for military personnel than for civilian personnel as reported by Casey et al [11].

Within active adverse event reporting systems, Frey et al [10] found at least 44.2% of their civilian subjects reporting post smallpox vaccination headaches. In a sample of mostly civilian subjects, Baggs et al [12] had 28.4% of subjects reporting headaches. With a mix of DoD civilian and military personnel, Olmstead et al [9] found 26.2% of subjects reporting headaches. Using only military subjects, Grabenstein & Winkenwerder [8] found 18.1% reporting headaches after smallpox vaccination. In other words, the larger the military composition of the subjects, the lower was the rate of reported headaches as post-vaccination adverse events. The fact that all of Frey et al's [10] subjects were primary vaccinees may account for part of the difference between the samples, according to Sejvar et al [15].

## Factual discrepancies or errors

The Committee on Smallpox Vaccination Program Implementation [16] reported that the national smallpox vaccination campaign had reached a cumulative total of 38,004 civilian smallpox vaccinations by July 25, 2003, with only 1,575 more civilian smallpox vaccinations over a year later, by July 31, 2004, for a total at that time of 39,579. Sejvar et al [15] reported 34,752 civilian smallpox vaccinations between January 24 and May 2, 2003. Casey et al [11] used 38,885 vaccinations as their baseline from January 24, 2003 to October 31, 2003, a number that matches closely with the Committee data [16]. Poland et al in a March 2003 report [17] indicated a total of 40,449 civilian smallpox vaccinations as of 13 February 2004 as well as a total of 730,580 military smallpox vaccinations as of 4 January 2005. However, Sejvar et al [1] reported 64,600 civilian smallpox vaccinations between December 16, 2002 and March 11, 2004, with apparently 24,151 additional smallpox vaccinations in less than four weeks between 14 February and 11 March 2004. While the time frame for civilian smallpox vaccinations was shorter in Sejvar et al [1] than that considered by the Committee [16], Sejvar et al [1] reported nearly 60% more civilian vaccinations that were otherwise officially reported, even though Dr. John Grabenstein co-authored at least two of the conflicting reports [1,17]. Sejvar et al [1] also reported considerably fewer military vaccinations than Poland et al [17], raising questions about the number of military vaccinations cited in Sejvar et al [1]. Furthermore, they added their 64,600 civilian vaccines to their 590,400 military vaccinees, obtaining a total of 665,000 vaccines, though their actual sum was short of 665,000 by exactly 10,000 subjects. Such errors are critical because they factored both 665,000 and 64,600 into their computations of incidence rates, as reported in their manuscript (see Table 2 in [1]), for not only headaches, but also for 18 other neurologic syndromes. It must be noted that both of the co-authors of the Sejvar et al [1] report who reviewed this comment admitted in their reviews that the 64,600 figure was incorrect and should have been approximately 40,000.

Furthermore, Sejvar et al [1] reported only 95 VAERS headache reports from among all 665,000 subjects, even though, using a far smaller data set including most of the same civilian subjects, Casey et al [11] had found at least 110 headaches reported as adverse events in VAERS. Sejvar et al [1] should have found at least as many headaches as Casey et al [11] because both their time frame and their number of civilian subjects were larger; in addition, they should have located many additional headache reports, given the percentage of headaches noted elsewhere by Olmstead et al [9] and by Grabenstein & Winkenwerder [8] among military personnel.

The scientific validity of the article by Sejvar et al [1] has probably been compromised by several errors. First, their estimate of 64,600 civilian vaccinations is much larger than the approximately 40,000 estimated from other sources concerning the 2002–2004 smallpox vaccination campaign [11,16,17], unless they were counting over 24,000 vaccinations provided that had not been officially reported along with those 40,000. Second, their addition of 64,600 civilian and 590,400 military vaccinees should have totalled to 655,000 rather than 665,000, unless there is an unexplained source of the additional 10,000 vaccinations. Third, of course, is that if the count of 64,600 civilians is incorrect, then the total should have been closer to 630,400 rather than 665,000. Fourth, it is not clear how Sejvar et al [1] counted only 95 headaches among VAERS reports for 665,000 subjects, when Casey et al [11], using the same civilian subjects but a total pool of subjects of less than 40,000, was able to identify a greater number (111) of headache reports in VAERS. Fifth, if their counting of VAERS headaches is incorrect and their total number of cases is incorrect, then their rates of a variety of adverse events are, by consequence, also incorrect. Essentially, such a large number of substantial errors or omissions can do little except to undermine the stated objective of Sejvar et al's [1] report, which was, as noted before, to "determine rates and describe the clinical features of neurologic events associated with smallpox vaccination," as well as raising questions about the factual validity of their conclusion that "During the 2002–2004 smallpox vaccination campaign, neurologic events were generally mild and self-limited, and no neurologic syndrome was identified at a rate above baseline estimates."

Given our observation that military samples, whether using active or passive reporting of adverse events following smallpox vaccination, tend to report fewer adverse events than civilian samples, their process of combining a relatively small civilian sample with a far larger military sample is also probably suspect in terms of generating accurate rates of adverse events, even if the numbers of civilian and military vaccines had been correct or were to be adjusted to correct values. The publishing of such uncorrected errors and weak methodologies by officials, most of whom are employed full-time by the U.S. government and/or the U.S. military, in pre-eminent medical journals, such as JAMA, does not contribute to public trust in the U.S. government's current approach to vaccination programs that were designed by public policy and law, at taxpayer expense, to support international efforts to counter the threat of bioterrorism. The scientific value of the research reported by Sejvar et al [1] has probably been compromised to such an extent that the article should either be retracted or the errors or omissions of fact and computation should be corrected throughout their report.

Despite logical protest by Grabenstein [6], Nass [5] appears to be correct in her assertion that military subjects have tended to underreport adverse events associated with smallpox vaccination, relative to civilian populations, as clearly shown in Table 1. Assuming that our assertions are valid with respect to smallpox vaccination within the U.S. military, underreporting of adverse symptoms associated with other controversial vaccinations, such as anthrax vaccination, is almost certain. Such underreporting has important implications for determining accurate risk-benefit ratios associated with vaccination [2-4]. Along with the types of errors presented here, uncertainty with respect to risk-benefit ratios weighs against making higher risk vaccinations mandatory, without informed consent, even for military service members, especially when there are legitimate research questions about adverse side effects of vaccination [3], including long-term effects [18]

## Competing interests

Dr. Schumm is a retired colonel, U.S. Army Reserve, and will receive retirement pay from the Department of Defense from January 2011 at age 60. Dr. Schumm has served as an expert witness for the plaintiffs in the Doe v. Rumsfeld civil suit, in which he primarily reanalyzed data from former studies of anthrax vaccine. Otherwise, Dr. Schumm has no financial interests in this research. He is not employed by any agency of the U.S. Department of Defense or the U.S. government, although he is an unpaid volunteer with the U.S. government program for "Employer Support of the Guard and Reserve" that encourages employers and Reserve Component soldiers to cooperate, in accordance with U.S. law, in allowing such soldiers to leave and return to their civilian employment without penalty.

## Authors' contributions

WRS is solely responsible for the content of this report. However, Dr. Meryl Nass and Lieutenant Colonel (Retired) John Richardson reviewed previous drafts of this report and suggested helpful improvements. As noted below, reviewers also provided helpful comments on previous drafts.

## Pre-publication history

The pre-publication history for this paper can be accessed here:



## Figures and Tables

**Table 1:** Variation in headache rates as a function of type of adverse event reporting and of type of sample

	Type of Adverse Event Reporting	
Type of Sample	Passive	Active
Military	< 0.016% [1]	18.1% [8]
Mixed Military and Civilian	0.014% [1]	26.2% [9]
Mostly Civilian	-	28.4% [12]
Civilian	0.283% [11]	44.2% [10]

Sources are indicated in brackets after each percentage.

## References

[B1] Sejvar JJ, Labutta RJ, Chapman LE, Grabenstein JD, Iskander J, Lane JM (2005). Neurologic adverse events associated with smallpox vaccination in the United States, 2002–2004. JAMA.

[B2] Neuzil KM, Griffin MR (2005). Vaccine safety: achieving the proper balance. JAMA.

[B3] Wortley PM, Schwartz B, Levy PS, Quick LM, Evans B, Burke B (2006). Healthcare workers who elected not to receive smallpox vaccination. Am J Preventive Medicine.

[B4] Quigley RB (2004). Uncertain benefit: the public policy of approving smallpox vaccine research. Am J Public Health.

[B5] Nass M (2003). Safety of the smallpox vaccine among military recipients (letter). JAMA.

[B6] Grabenstein JD, Riddle JR, Arness MK, Winkenwerder W (2003). In reply to "Safety of the smallpox vaccine among military recipients". JAMA.

[B7] Hilleman MR (1996). Cooperation between government and industry in combating a perceived emerging pandemic. JAMA.

[B8] Grabenstein JD, Winkenwerder W (2003). US Military Smallpox Vaccination Program experience. JAMA.

[B9] Olmsted SS, Grabenstein JD, Jain AK, Comerford W, Giambo P, Johnson P, Mopsik J, Zimmerman SR, Lurie N (2005). Use of an electronic monitoring system for self-reporting smallpox vaccine reactions. Biosecurity and Bioterrorism: Biodefense Strategy, Practice, and Science.

[B10] Frey SE, Couch RB, Tacket CO, Treanor JJ, Wolff M, Newman FK, Atmar RL, Edelman R, Nolan CM, Belshe RB (2002). Clinical responses to undiluted and diluted smallpox vaccine. N Engl J Med.

[B11] Casey CG, Iskander JK, Roper MH, Mast EE, Wen XJ, Torok TJ, Chapman LE, Swerdlow DL, Morgan J, Heffelfinger JD, Vitek C, Reef SE, Hasbrouck LM, Damon I, Neff L, Vellozzi C, McCauley M, Strikas RA, Mootrey G (2005). Adverse events associated with smallpox vaccination in the United States, January-October 2003. JAMA.

[B12] Baggs J, Chen RT, Damon IK, Rotz L, Allen C, Fullerton KE, Casey C, Nordenberg D, Mootrey G (2005). Safety profile of smallpox vaccine: insights from the Laboratory Worker Smallpox Vaccination Program. Clin Inf Diseases.

[B13] Halsell JS, Riddle JR, Atwood JE, Gardner P, Shope R, Poland GA, Gray GC, Ostroff S, Eckart RE, Hospenthal DR, Gibson RL, Grabenstein JD, Arness MK, Tornberg DN (2003). Myopericarditis following smallpox vaccination among vaccinia-naïve US military personnel. JAMA.

[B14] Talbot TR, Bredenberg HK, Smith M, LaFleur BJ, Boyd A, Edwards KM (2003). Focal and generalized folliculitis following smallpox vaccination among vaccinia-naïve recipients. JAMA.

[B15] Sejvar J, Boneva R, Lane JM, Iskander J (2005). Severe headaches following smallpox vaccination. Headache.

[B16] Committee on Smallpox Vaccination Program Implementation, Institute of Medicine (2005). The Smallpox Vaccination Program.

[B17] Poland GA, Grabenstein JD, Neff JM (2005). The US smallpox vaccination program: a review of a large modern era smallpox vaccination implementation program. Vaccine.

[B18] Schumm WR, Jurich AP, Bollman SR, Webb FJ, Castelo CS (2005). The long term safety of anthrax vaccine, pyridostigmine bromide (PB) tablets, and other risk factors among Reserve Component veterans of the first Persian Gulf War. Medical Veritas.

